# κ-Carrageenan Hydrogel as a Matrix for Therapeutic Enzyme Immobilization

**DOI:** 10.3390/polym14194071

**Published:** 2022-09-28

**Authors:** Olga N. Makshakova, Liliya R. Bogdanova, Anastasiya O. Makarova, Aleksandra M. Kusova, Elena A. Ermakova, Mariia A. Kazantseva, Yuriy F. Zuev

**Affiliations:** 1 Kazan Institute of Biochemistry and Biophysics, FRC Kazan Scientific Center of RAS, Lobachevsky St., 2/31, 420111 Kazan, Russia; 2 HSE Tikhonov Moscow Institute of Electronics and Mathematics, Tallinskaya St., 34, 123458 Moscow, Russia

**Keywords:** polysaccharides, nanogels, enzymes, immobilization, delivery systems

## Abstract

During the last few decades, polysaccharide hydrogels attract more and more attention as therapeutic protein delivery systems due to their biocompatibility and the simplicity of the biodegradation of natural polymers. The protein retention by and release from the polysaccharide gel network is regulated by geometry and physical interactions of protein with the matrix. In the present work, we studied the molecular details of interactions between κ-carrageenan and three lipases, namely the lipases from *Candida rugosa*, *Mucor javanicus*, and *Rhizomucor miehei*—which differ in their size and net charge—upon protein immobilization in microparticles of polysaccharide gel. The kinetics of protein release revealed the different capability of κ-carrageenan to retain lipases, which are generally negatively charged; that was shown to be in line with the energy of interactions between polysaccharides and positively charged epitopes on the protein surface. These data create a platform for the novel design of nanocarriers for biomedical probes of enzymatic origin.

## 1. Introduction

The promising innovative biomaterials for drug delivery are hydrogels based on natural biopolymers (proteins and polysaccharides) [1,2,3,4]. It is a consequence of their biocompatibility, the similarity of their structure and physicochemical properties with human extracellular matrix. More technologically promising are “physical” hydrogels, typical for natural biopolymers, in which a three-dimensional polymer network exists due to the mechanical weave of polymer molecules and/or stabilization by intermolecular interactions, including ionic bridges, hydrogen bonding, and hydrophobic interactions [5]. The polysaccharide-based nanomaterials, such as mesoporous hydrogels, display original physicochemical and biological properties, providing a method for novel biomedical applications, such as drug delivery [6,7], tissue engineering and regenerative medicine [8,9], biosensing and molecular diagnostics [10], tissue imaging and therapy [11]. Natural poly-saccharides show low toxicity and improve biocompatibility and stability under physiological conditions for the engineered materials. The present work extends our recent results on polysaccharide-based systems for immobilization and carriers of enzymes as a potential drug delivery system.

The polysaccharide used in the present study to prepare hydrogels, is kappa-carrageenan (κ-carrageenan), a linear anionic polymer extracted from red seaweed (*Rhodophyta*). It is a non-toxic and biodegradable biopolymer, which possesses anti-viral activity [12], which serves for tasks of tissue engineering [13] and drug delivery [14]. κ-carrageenan consists of galactose and (3,6)-anhydrogalactose repeat units connected by alternating α-(1,3)- and β-(1,4)-glycosidic links and bears one sulfate group per galactose at the O4 position. It forms gel in the presence of salts at ambient temperatures. The admixture of proteins may assist κ-carrageenan gelation [2,5]. 

Depending on the intrinsic chemical structure of polysaccharides and protein, their mixing can result in the coupling or phase-separation of components. Both local mixing and demixing behaviors are mainly electrostatically driven. Proteins, possessing a positive net charge, form the coacervates with anionic polysaccharides. As we recently demonstrated on the example of lysozyme and κ-carrageenan coacervates [15], such interactions significantly modify the protein structure during the formation of bicomponent junctions and the common protein–polysaccharide structure [16]. On the other hand, the negatively charged globular proteins, trapped in the matrix of anionic polysaccharides upon the fast termination of the gelation process, usually retain their functional structure [2]. The release of protein from the polysaccharides matrix is determined by its confined diffusion in the gel, by the ratio of the mesh size of the gel and protein dimension, and by intermolecular interactions of the protein with the polysaccharide matrix [17]. Since both the poly-saccharide chains and the protein surface usually have patterns with different physicochemical properties, their interactions and protein release are strongly case-dependent. 

In the present study, we considered a set of lipases with differences size, net charge (general negative), and surface topology. Lipases, triacylglycerol hydrolases, are lipolytic enzymes with potential therapeutic activity. Our previous experience rests in the engineering of various colloid lipase-based systems [18]. Additionally, lipases are actively used in the impairment therapy of specific lipid metabolic disorders, such as insulin resistance, obesity, and metabolic syndrome [19]. The peculiar functional property of lipases to operate in the presence of a definite interface (e.g., lipid bilayer) generates different technological applications, which use enzyme immobilization, including the polysaccharide matrix [20]. 

In the present work, we applied a set of complementary research to obtain novel information on the properties of the polysaccharide-based drug delivery systems for lipases as a model of nanocarrier agents. We performed the analysis of interactions between κ-carrageenan and three lipases, namely the lipases from *Candida rugosa*, *Mucor javanicus*, and *Rhizomucor miehei*. The combination of spectrophotometric experiments and computer modeling was applied to rationalize the aspects of intermolecular interactions and the release of lipases from κ-carrageenan gels. The varying efficiencies of lipase immobilization in polysaccharide gels will help the rational design of nanocarriers for biomedical probes of enzymatic origin. 

## 2. Materials and Methods

### 2.1. Materials

Anionic polysaccharide κ-carrageenan Type I was purchased from Sigma-Aldrich. This polysaccharide, with a viscosity-average molecular weight of M_η_ = 430 kDa, was used without additional purification. The K^+^, Ca^2+^, and Na^+^ contents in the polysaccharide sample were below 6, 1, and 1 wt. %, correspondingly. Three types of lipase (triacylglycerol hydrolase EC 3.1.1.3) were used: lipase *Candida rugosa* (L1754, Sigma-Aldrich, St. Louis, MI, USA), molecular weight 57,094 Da, pI = 4.7; lipase *Mucor javanicus* (L8906, Sigma-Aldrich, St. Louis, MI, USA), molecular weight 43,419 Da, pI = 6.3; lipase *Rhizomucor miehei* (L4277, Sigma-Aldrich, St. Louis, MI, USA), molecular weight 39,576 Da, pI = 4.9. 

We used medical ethanol (95%, Rosbio, St Petersburg, Russia), calcium chloride (chemically pure, Tatkhimprodukt, Kazan, Russia), and 2-amino-2-hydroxymethylpropane-1,3-diol (Tris, analytical grade, Diaem, Moscow, Russia). To prepare all solutions, water purified with an “Arium mini” ultrapure water system (“Sartorius”, Göttingen, Germany) was used. Buffer solutions were prepared with tris(hydroxymethyl)aminomethane (hereinafter Tris), produced by Helicon, and hydrochloric acid (Tatchemproduct, Russian Federation).

### 2.2. Preparation of Initial Solutions and Gels

The stock solution of κ-carrageenan (1.6 wt. %) was prepared by dissolving the corresponding polysaccharide samples in Tris-HCl buffer (50 mM pH = 7.4) at 70 °C. The enzyme solution (0.5 mL) was added to 0.5 mL of a concentrated polysaccharide solution. The resulting solutions were sonicated at 35 kHz with a Bandelin SONOREX TK52 ultrasonic bath (Germany, 100 W) for 30 min at 35 °C and were used to prepare hydrogels. The concentration of κ-carrageenan in the resulting solutions was 0.8 wt. % and the concentration of lipases was 6.5 × 10^−5^ M.

To prepare hydrogel spheres, 0.5 mL of a solution of κ-carrageenan with lipase was added dropwise with constant stirring (500 rpm) to 1.5 mL of a 1 M solution of calcium chloride using a medical syringe with a needle diameter of 0.63 mm. The salt solution was pre-cooled to 4 °C. The prepared microspheres were left in the salt solution for 10 min and then the spheres were washed as follows: the solution was poured from the vessel with the formed spheres using a dispenser, then 1 mL of Tris-HCl buffer was added to the spheres and thoroughly mixed, and then the water phase was drained again. This procedure was carried out 2 times to wash out the protein from the surface of microspheres. 

### 2.3. Enzyme Release from Hydrogel

To assess the holding capacity of the hydrogel, the release of enzymes from κ-carrageenan hydrogel spheres was studied using a Lambda 25 spectrophotometer with a temperature-controlled cuvette compartment. Five spheres were placed into polypropylene microtubes (Eppendorf, Axygen, Union City, CA, USA), buffer was added to attain a volume of 2 mL, and they were kept for adjusted time intervals. After a given time interval, the aliquots of solution were taken for spectrophotometric determination of the concentration of enzymes released from the hydrogel spheres into the solution.

The concentration of released enzymes was determined at 23 °C by absorption at λ = 280 nm. Enzyme extinction coefficients (https://www.protpi.ch/Calculator/ProteinTool (accessed on 8 August 2022)) were: 37,000 M^–1^cm^–1^ (*Candida rugosa*), 44,810 M^–1^cm^–1^ (*Mucor javanicus*), and 48,820 M^–1^cm^–1^ (*Rhizomucor miehei*). The equation

(1) $$Y=\frac{m_{t}}{m-m_{0}}\cdot 100\%$$

was used to determine the probe release (*Y*), where *m_t_* is the mass of protein in the solution at time *t*, *m*_0_ is the mass of protein that remained unencapsulated, and *m* is the initial protein mass. The immobilizing efficiency (*F*) of the enzyme in spheres was determined:

(2) $$F=\frac{m-m_{0}}{m}\cdot 100\%$$

The results presented below are the averaged values from three measurements.

### 2.4. Enzyme Activity

Experiments were carried out on the Lambda 25 spectrophotometer (Perkin Elmer, Waltham, MA, USA) with a temperature-controlled cell compartment. To determine the activity of lipases, the p-nitrophenyl laurate was used as a substrate. Enzyme activity was assayed by measuring the amount of p-nitrophenol (p-NP) released from the substrate (λ = 400 nm, ε = 12,700 M^−1^cm^−1^, determined in an independent experiment).

### 2.5. Computer Simulations 

#### 2.5.1. Initial Protein Structures

The protein structures were taken from protein databases: lipase from *Candida Rugosa*, pdb code 1trh; lipase from *Rhizomucor miehei*, pdb code 6qpp. The missing loops were constructed and refined with the Modeller 9.15 approach [21]. 

The structure of lipase from *Mucor javanicus* was built using the homology modeling approach. The primary structure of the protein was taken from [22]. Three programs for homology modeling were used, including Phyre2 [23], Swiss-Model [24], and I-Tasser [25]. Then models were ranked according to the Q-mean values [24] and the model with the highest Q-mean value (obtained by I-Tasser program) was refined with a short MD simulation of protein in a water box with Charmm36 force field [26] and Gromacs program [27].

#### 2.5.2. Molecular Docking of κ-Carrageenan Fragments to Lipases

The molecular docking procedure was performed using the Autodock4.2 program [28]. As the protein ligand, the κ-carrageenan trisaccharide was built, which was successfully used in our previous work in the framework of fragment-based docking [4,15]. Ten runs for each protein were performed and 100 most favorable binding poses were analyzed. First, the resulting binding poses were filtered to meet the conditions of the values of trisaccharide torsion angles in the allowed regions [5]. Second, the poses were clustered with an RMS cluster tolerance of 10 Å and the most populated clusters possessing favorable energy were analyzed in respect to molecular contacts. 

The energy of polysaccharide–protein binding is:

(3) $$E=E_{vdW}+E_{elec}+E_{HB}+E_{desolv}+E_{entr}$$

where *E_vdW_* is the energy of van der Waals protein–ligand interactions, *E_elec_* is the energy of electrostatic interactions, E_*HB*_ is energy of hydrogen bonds, *E_desolv_* characterizes the desolvation energy, and *E_entr_* is the contribution from the conformational entropy of the ligand.

#### 2.5.3. Protein Net Charge Calculations

The protein net charge was calculated on the primary amino acid sequence using Protpi (https://www.protpi.ch/Calculator/ProteinTool (accessed on 8 August 2022)). The protein electrostatic surface potentials were determined with the Adaptive Poisson–Boltzmann Solver (APBS) [29] web service (https://server.poissonboltzmann.org/ (accessed on 8 August 2022)) The pH was set as 7.4 The protein electrostatic surface potential visualization was carried out using the UCSF Chimera software [30].

## 3. Results and Discussion

**Figure 1:**
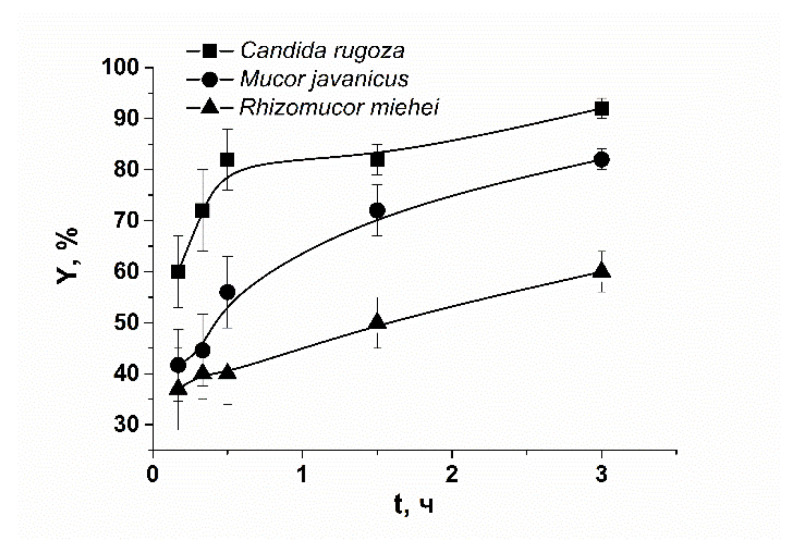
Lipase release from κ-carrageenan-based hydrogel microspheres.

Our testing of protein binding with polysaccharide hydrogel showed that lipases immobilize rather well in the κ-carrageenan matrix (Figure 1). It was revealed that the high percentage of enzymes remain in hydrogel microcapsules for several hours with the immobilizing efficiency of 25, 35, and 37% for lipases *Candida rugosa* (CR), *Mucor javanicus,* (MJ) and *Rhizomucor miehei* (RM), correspondingly. The analysis of protein release curves showed that the largest CR lipase has the fastest release. This means that the protein size and mesh size of the polysaccharide network are not decisive for this process. The quantitative estimation of protein activity highlighted that protein remains catalytically functional in the immobilized state or after immobilization, but we cannot distinguish the investment from entrapped and released species and do not further discuss these characteristics here. 

**Figure 2:**
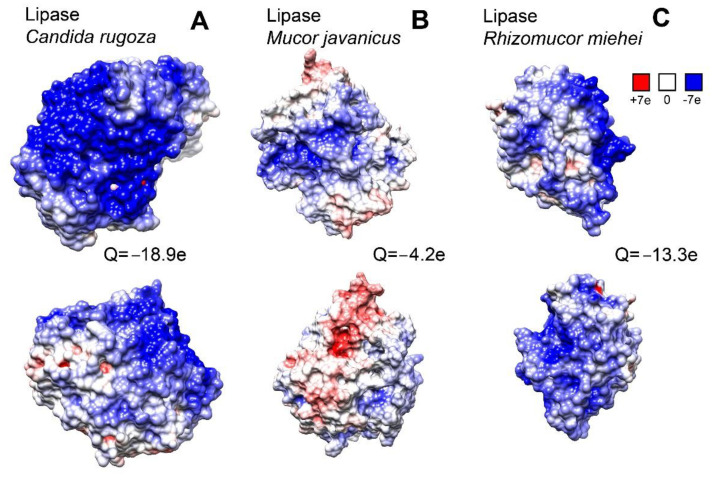
The surface charge simulation of lipase from *Candida* (**A**), lipase from *Mucor javanicus* (**B**), and lipase from *Rhizomucor miehei* (**C**). For every protein, the view of opposite surface is shown above and below.

The CR lipase has the largest net charge among the three lipases studied (Figure 2). This may affect the electrostatic repulsion between the lipase molecules and the negatively charged κ-carrageenan matrix and result in a faster release of enzymes. 

The surface electrostatic potential significantly varies from protein to protein in the set of lipases. This difference in protein net charge and surface distribution may influence the protein–polysaccharide interactions. For short range contacts, a number of weak physical interactions, such as van der Waals forces and hydrogen bonds, become important. Furthermore, with the molecular docking experiment, we analyzed how the protein surface topology can influence the polysaccharide binding. 

**Figure 3:**
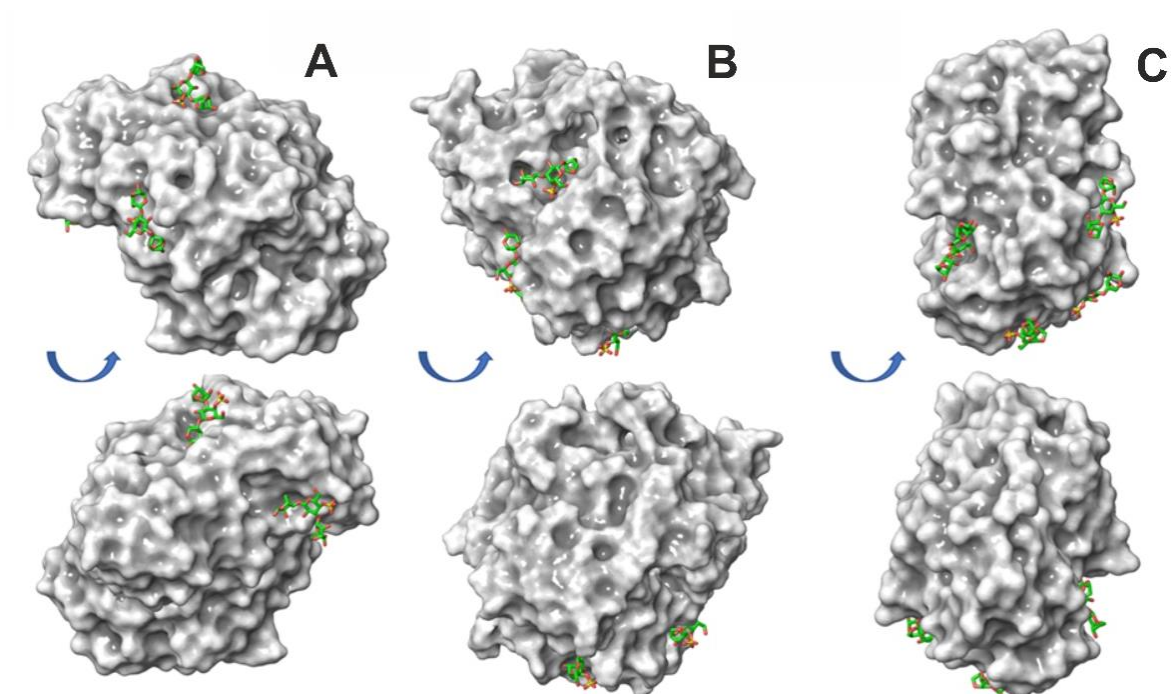
Most probable sites for κ-carrageenan binding with lipases from *Candida rugosa* (**A**), *Mucor javanicus,* (**B**) and *Rhizomucor miehei* (**C**). Proteins are shown in the surface representation and ligands in the stick representation.

To determine the protein surface epitopes for κ-carrageenan binding, fragment-based molecular docking was performed. In this approach, a natural long chain of polysaccharide was divided into short representative fragments, then these short fragments were docked to protein molecules. This procedure allows a decrease in the extremely high conformational variability of the polymer chain, which is typical for long polysaccharides and impossible to grasp in silico. The docking poses of short polysaccharide oligomers indicate the most probable sites of polysaccharide–protein binding and, at certain conditions, may help to trace the orientation of real polysaccharides in respect to protein molecules. Earlier, the trisaccharide fragment was shown to be efficient at tackling the binding sites of oligomers for up to dozens of sugar residues (e.g., 24 residues of κ-carrageenan can interact with one lysozyme molecule) [4,15]. However, in the three lipases studied in this work, the docking poses of the κ-carrageenan trisaccharide were rather isolated (Figure 3). This implies that it is unlikely that those rather extended epitopes are capable of binding longer polysaccharide fragments determined for lysozyme [4,15] and binase [4]. In the lipases studied here, the interactions with the κ-carrageenan chain are rather stabilized by the point contacts with short polysaccharide fragments, which are up to three saccharide residues. 

All three lipases have four of the most populated binding sites with favorable energies of κ-carrageenan binding that can be considered to be true positive hits. These hits were determined according to their localization in the upper left corner of the plot for the cluster population vs. binding energy after the clustering of the docking poses. The cut-off, dividing the ‘true positive’ hits from ‘false positive’ ones, was related to the cluster population with *n* = 7. Due to this division, we considered only the hits with the highest population, mostly represented by *n* from 9 to 18, and discarded the low-populated clusters, mostly represented by *n* from 1 to 4. For each protein studied, the population of the four ‘true positive’ hits covered about 50% of the total population. The energies (calculated for trisaccharide) in the clusters are within the range of −3.6–−2 kcal/mol for CR lipase, −4.0–−3.5 kcal/mol for MJ lipase, and −4.2–3.5 kcal/mol for RM lipase. In the CR lipase, the clusters reside in the sites around the positively charged residues Lys67, Lys 217, Arg250, and Lys244. In the MJ lipase, the κ-carrageenan fragments interacted with Lys146, Arg288, Lys265, and Lys295 residues. In the RM lipase, the binding sites were located at Arg124, Lys147, Arg162, Lys167, Arg180, and Lys231 residues. The examples of binding pose clusters possessing the most favorable interaction energies are shown in Figure 3.

The variation of interaction energies between lipases and κ-carrageenan trisaccharide revealed that, for the CR lipase, the interactions with the κ-carrageenan fragment is systematically weaker than those for other lipases. The affinity of lipases to trisaccharide increases in the order: *Candida Rugosa* -> *Mucor javanicus* -> *Rhizomucor miehei*. Speculating about the binding of long polysaccharide chains (i.e., in our experiment, one protein molecule was exposed to ~200 trisaccharide units) to any of the proteins studied, the total interaction energy of the protein with the polysaccharide is more favorable than with a trisaccharide due a larger number of such contacts to one protein, which can be formed either by one κ-carrageenan chain or by several polysaccharide chains. However, since the interactions in each binding site are systematically weaker for CR lipase, one can anticipate that the binding of a long polysaccharide chain to this protein is also weaker than to the other two. 

Summing up, the interaction energies being in line with the experimental values of the efficiency of encapsulation may imply that the efficiency of encapsulation is determined by the physical contacts between the protein and the polysaccharide.

Summing up the results of molecular docking, the interaction energies being in line with the experimental values of the efficiency of encapsulation may imply that the efficiency of encapsulation is determined by the physical contacts between the protein and the polysaccharide. It seems that different lipases give differing responses upon their immobilization depending on the molecular details of interactions with the polysaccharide.

Immobilization includes all aspects of protecting and stabilizing the active enzyme molecules from such external unfavorable factors, such as heat, redox potential, shear, temperature, light, oxygen, moisture, etc. The controlled release can facilitate the delivery of the immobilized material to the target site while keeping its optimal activity [31]. The important parameters to determine the possibility of the controlled release of the immobilized substance from the polymer matrix are the molecular weight and charge of the probe molecule [32,33]. 

This work shows that the structure and properties of an immobilized protein and its host polysaccharide matrix determine the effectivity of enzyme immobilization and release rate, as well as the level of its enzymatic activity. We show that the functional activity of immobilized enzymes can remain on a rather high level and the studied κ-carrageenan hydrogels present a smart system for enzyme encapsulation.

## 4. Conclusions

The present work is devoted to a comprehensive study of properties of the polysaccharide-based drug delivery system as nanocarrier for biomedical probes of enzymatic origin, namely several lipases differing in molecular structure and surface charge. We used the κ-carrageenan hydrogels, which supports its spatial structure and intrinsic properties by a variety of physical crosslinks including ionic bridges, hydrogen bonding, and hydrophobic forces, as potential delivery systems for enzymatic therapy. These materials represent a biopolymer matrix, which can be successfully used for immobilization of bioactive enzymes, altering their activity and controllable release. This work shows that the structure and properties of immobilized protein and its host polysaccharide matrix determine the effectivity of enzyme immobilization and release rate, as well as the level of its enzymatic activity. We show that the functional activity of immobilized enzymes can remain on a rather high level and the studied κ-carrageenan hydrogels present a smart system for enzyme immobilization.

The obtained results show the possibility to control the action time of immobilized enzymes to engineer the corresponding drugs. Evidently, the polymer matrix may be of different origin with differing retention times and release efficiencies. Looking at other candidates for the role of enzyme carrier, it is interesting to analyze the mechanisms of protein–polysaccharide binding.

## Data Availability

The data in this study are available upon reasonable request from the corresponding author.
